# Estimating the Global Public Health Implications of Electricity and Coal Consumption

**DOI:** 10.1289/ehp.119-821

**Published:** 2011-02-21

**Authors:** Julia M. Gohlke, Reuben Thomas, Alistair Woodward, Diarmid Campbell-Lendrum, Annette Prüss-üstün, Simon Hales, Christopher J. Portier

**Affiliations:** 1 Laboratory of Molecular Toxicology, National Institute of Environmental Health Sciences, Research Triangle Park, North Carolina, USA; 2 School of Population Health, University of Auckland, Auckland, New Zealand; 3 Department of Public Health and Environment, World Health Organization, Geneva, Switzerland

**Keywords:** air pollution, climate change, coal, electricity, energy policy, global health, health impact modeling, infant mortality, life expectancy, time series

## Abstract

Background: The growing health risks associated with greenhouse gas emissions highlight the need for new energy policies that emphasize efficiency and low-carbon energy intensity.

Objectives: We assessed the relationships among electricity use, coal consumption, and health outcomes.

Methods: Using time-series data sets from 41 countries with varying development trajectories between 1965 and 2005, we developed an autoregressive model of life expectancy (LE) and infant mortality (IM) based on electricity consumption, coal consumption, and previous year’s LE or IM. Prediction of health impacts from the Greenhouse Gas and Air Pollution Interactions and Synergies (GAINS) integrated air pollution emissions health impact model for coal-fired power plants was compared with the time-series model results.

Results: The time-series model predicted that increased electricity consumption was associated with reduced IM for countries that started with relatively high IM (> 100/1,000 live births) and low LE (< 57 years) in 1965, whereas LE was not significantly associated with electricity consumption regardless of IM and LE in 1965. Increasing coal consumption was associated with increased IM and reduced LE after accounting for electricity consumption. These results are consistent with results based on the GAINS model and previously published estimates of disease burdens attributable to energy-related environmental factors, including indoor and outdoor air pollution and water and sanitation.

Conclusions: Increased electricity consumption in countries with IM < 100/1,000 live births does not lead to greater health benefits, whereas coal consumption has significant detrimental health impacts.

Developing energy policies that improve global health requires understanding the complex interplay between systems for energy delivery and sustainable, healthy human environments. Access to a clean, dependable, and affordable energy source is a prerequisite for good health (Modi et al. 2005). Electricity may be used to power a reliable water and sanitation infrastructure and reduce exposure to indoor air pollution from relatively dirty energy sources such as coal and wood burning in homes. The increasing and potentially irreversible health risks associated with greenhouse gas emissions have resulted in a global call for the development of new energy policies that emphasize efficiency and low-carbon energy sources (Haines et al. 2007, 2009; Markandya et al. 2009; Wilkinson et al. 2007).

International comparisons of energy consumption per capita with national life expectancy (LE) indicate a positive association, and with infant mortality (IM), a negative association, particularly at lower levels of consumption (Wilkinson et al. 2007); these associations represent cross-sectional, ecological comparisons. It is difficult to tease apart the effect of energy use in a household and the indirect health gains from economic development supported by energy use. To complicate matters further, clear relationships among economic growth, energy consumption, and LE are not fixed, as shown by the experience of countries such as Japan, where health statistics improved before progress in economic indicators (Riley 2007; United Nations Environmental Programme 2007).

Access to a centralized power source is necessary to gain many of the benefits of clean power. However, depending on the way power is generated, new risks may be introduced that are not reflected in the market price, often referred to as external costs. The social and environmental external costs of a centralized power source have been estimated using a life-cycle analysis approach (Bickel and Friedrich 2005; Oak Ridge National Laboratory 1995; Spath et al. 1999). Public health impacts dominate the costs, accounting for > 70% of the estimated external costs for fossil fuel–based power generation. Direct health impacts associated with emissions of classic air pollutants [particulate matter (PM), sulfur oxides, nitrous oxides, volatile organic compounds, carbon monoxide, and ozone] during the power generation stage account for most of the external costs associated with fossil fuel–based power generation today. The most recent analysis of externalities in energy production and use completed for the United States by the National Academies of Sciences (NAS) suggests that the total costs added up to more than $120 billion in 2005 (National Research Council 2010).

The NAS report and other investigators (Bickel and Friedrich 2005) have also estimated the climate-related external costs of energy technologies, which include health, environmental, security, and infrastructure impacts. For coal and transportation fuels, the costs associated with climate-related damages exceeded other (nonclimate-related) impacts when the assumed marginal climate damage was > $30 U.S. dollars per ton carbon dioxide equivalent (CO_2_-eq) in 2005 (National Research Council 2010). The estimated climate-related damages per ton of CO_2_-eq for 2030 were 50–80% higher than those for 2005. The Externalities of Energy (ExternE) project estimated that approximately 25% of the external costs of fossil fuel–powered generation systems are due to climate change–related impacts from emissions of CO_2_, methane, and nitrogen dioxide (Bickel and Friedrich 2005). Of these costs, > 95% are accounted for by health impacts, including those related to thermal extremes, increased incidence of malaria, diarrheal disease, and malnutrition (Rabl and Spadaro 2006; Rabl et al. 2007).

These figures are a reminder that health and energy are closely linked, yet health has seldom been a focus in energy policy research related to climate change mitigation (Creyts et al. 2007; Stern 2006). Energy needs differ—some populations currently may have too little energy to achieve good health; others may benefit, in health terms, by reducing their levels of consumption (Markandya et al. 2009). One approach to mitigation divides responsibility based on the proportion of “high emitters” in each nation. It suggests a minimum level of individual CO_2_ emissions to protect those who do not yet have adequate access to electricity (Chakravarty et al. 2009).

The primary emphasis of the present analysis was to compare health impacts of electricity consumption from two perspectives using three complementary sets of data. First, we analyzed time-series data sets on health and energy statistics from 1965 to 2005 to determine the extent and reliability of the relationship between LE or IM and electricity consumption across 41 countries with diverse development trajectories. Next, we compared results with bottom-up approaches that estimate health impacts via exposure modeling and use of specific exposure–disease outcome relationships established in the literature. We looked at the World Health Organization (WHO) Environmental Burden of Disease (EBoD) estimates (Prüss-Üstün et al. 2003) for ambient air pollution, indoor air pollution, and water and sanitation in each of these countries with the goal of determining relationships between electricity and coal consumption and more specific health impacts related to power generation. Finally, we compared our results with those of an application of the Greenhouse Gas and Air Pollution Interactions and Synergies (GAINS) model (Amann et al. 2008) to estimate air pollutant emissions from coal-fired power plants, consequent human exposure to PM, and the potential life-shortening effect of this exposure. Our aim was to assist the development of new ways to compare the positive and negative health impacts of power generation in widely varying populations.

## Materials and Methods

*Data.* LE, IM, electricity use, coal consumption, and population data between the years of 1965 and 2005 were obtained from the Gapminder database (Rosling 2009). The data sets were derived from several sources: UNICEF statistics (Hill et al. 2006) for IM, defined as the number of deaths of infants < 1 year of age per 1,000 births; the Human Mortality Database (Wilmoth and Shkolnikov (2009); and World Population Prospects (United Nations Population Division 2009) for LE at birth, World Development Indicators Online (World dataBank 2009) for electric power consumption per capita, and Statistical Review of World Energy (British Petroleum 2009) for coal consumption per capita. Of the 200 countries represented in the IM and LE data sets, 41 had adequate electricity and coal consumption data for the 1965–2005 time span. The LE, IM, electricity, and coal consumption data sets for the 41 countries are described in greater detail in the Supplemental Material, “Methods–Data Description,” and plotted in Supplemental Material, Figures 1–4 (doi:10.1289/ehp.1002241).

**Figure 1:**
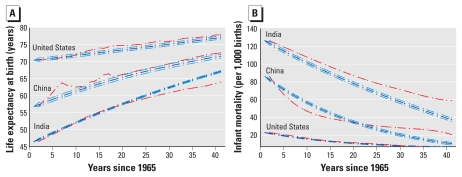
Time-series AR model results for LE at birth (*A*) and IM (*B*) in the United States, China, and India, representing the highest population countries in the low-IM/high-LE, mid-IM/LE, and high-IM/low-LE groups, respectively. LE at birth (years) and IM (per 1,000 live births) are plotted in red; results of the model described by Equation 1, including 95% confidence intervals, are plotted in blue. Adjusted *R*^2^ values are 0.92 (India), 0.74 (China), and 0.66 (United States) for LE models and 0.79 (India), 0.87 (China), and 0.92 (United States) for IM models.

*Autoregressive models for low-, mid-, and high-IM countries.* Autoregressive (AR) time-series models are commonly used to model LE and IM, particularly when there are insufficient data for all potential explanatory factors (Antunes and Waldman 2002; El-Zein et al. 2004; Kale et al. 2004; Kovats et al. 2004; Levine et al. 2001).

We modeled LE or IM using the following AR equation for each country:

*y*(*t*) = *a*_0_ + *a*_1_*u*_1_(*t*) + *b*_1_*u*_2_(*t*) + *dy*(*t*–1) + *e*(*t*), [1]

where *y*(*t*) is the average LE or IM at time *t* (years or mortality per 1,000 births), *u*_1_(*t*) is the average coal consumption per capita at time *t* (kilowatt hour per person per year), *u*_2_(*t*) is the average electricity consumption per capita at time *t* (kilowatt hour per person per year), *y* is the previous year time point (*t* – 1) and *d* is the coefficient of this parameter, *e*(*t*) is the zero mean normally distributed noise, and *a*_1_ and *b*_1_ are the coefficients being estimated. Equation 1 can be expanded to separate the dependencies of LE or IM solely due to patterns of coal and electricity consumption [see Supplemental Material, “Methods–AR Model Description” [doi:10.1289/ehp.1002241)]. The model was applied to individual country data sets. IM and LE data between the years of 1965 to 2005 were plotted against model results incorporating electricity use per capita and coal consumption per capita for each country (see Supplemental Material, Figure 5 (doi:10.1289/ehp.1002241)].

The individual countries were grouped into three categories, based on tertiles of the empirical joint probability distributions of IM and LE of all countries in the data set for the year 1965: countries with IM between 105 and 156 per 1,000 live births and LE between 44 and 57 years of age in 1965 (high IM/low LE), countries with IM between 44 and 98 per 1,000 births and LE between 56 and 70 years of age in 1965 (mid-IM/LE), and countries with IM between 14 and 39 per 1,000 births and LE between 69 and 71 years of age in 1965 (low IM/high LE). For each of the three groups, a composite model was developed where the individual country contribution to parameter fits of the composite model was given equal weight. To find the model that best fitted the group of countries across all the time points, parameter estimates were generated using the least squares approach on the model given by Equation 1.

*Analysis of cross-sectional WHO environmental burden of disease reports.* The EBoD series estimates the attributable fraction of disease due to a particular environmental risk factor using the general framework for global assessment described in the *The World Health Report 2002—Reducing Risks, Promoting Healthy Life* (WHO 2002). Individual reports on a specific environmental risk factor first outline the evidence linking the risk factor to health and then describe a method for estimating the health impact of that risk factor on the population. Only relationships between exposure and disease that were sufficiently well described to permit quantitative estimates of the disease burden are considered in these reports. Risk factors with long latency periods or nonspecific outcomes, factors with exposures that are difficult to assess at the population level, and factors that are distal to the outcomes are particularly difficult to quantify (Prüss-Üstün et al. 2003). To date, WHO has assessed 16 environmental risk factors worldwide. Results from the reports for outdoor air pollution (Cohen et al. 2005), indoor air pollution (Desai et al. 2004), and water and sanitation (Fewtrell et al. 2007). These reports estimated the total burden of disease attributable to each of the environmental factors in 2002. We then looked at the relationships between attributable disease burden for each of these three environmental factors individually, as well as the disease burden attributable to combinations of the factors, in each country based on the WHO reports against per capita electricity and coal consumption in 2002. Linear correlation between these two data sets was then tested using the corr function in Matlab (MatLab, Natick, Massachusetts, USA).

*Analysis using the GAINS model.* We used the GAINS model (Amann et al. 2008; Markandya et al. 2009), an integrated model estimating air pollutant emissions from coal-fired power plants, consequent human exposure to PM, and the potential life-shortening effect of this exposure, for three regions: the European Union, India, and China. The GAINS model is described in more detail in the Supplemental Material, “Methods–GAINS Model Description” (doi:10.1289/ehp.1002241).

To compare the results from the GAINS model with results from the AR model described above, results from the AR model were translated into comparable units. The GAINS model results are expressed in years of life lost (YLL) over the lifetime of a cohort of adults > 30 years of age, using dose–response estimates of premature mortality identified in adults (Pope et al. 1995). Results from the AR model coefficients are expressed in terms of change in LE or IM per 1,000 kWh per capita. Therefore, the coal consumption coefficients (*a*), as described in Table 1, were multiplied by the average coal consumption per capita in 2005 (the year in which the GAINS model is applied) for the European Union (low-IM/high-LE model), China (mid-IM/LE model), and India (high-IM/low-LE model), respectively. To match the units expressed in the GAINS model results, the time-series AR results were multiplied by the average LE in 2005 in the European Union, India, and China. An alpha level of 0.05 defined statistical significance.

**Table 1:** Model parameter estimates (mean and 95% confidence limit) for LE and IM predicted for the three groups of countries in 1965.

Model parameter	High IM/low LE^*a*^	Mid-IM/LE^*b*^	Low IM/high LE^*c*^
IM (per 1,000 births)
Intercept (*a*_0_) change in IM per year	–0.46 (–0.97 to 0.05)	–0.397 (–0.657 to –0.137)*	–0.04 (–0.09 to 0.01)
Electricity coefficient (*b*_1_)^*d*^	–0.66 (–1.02 to –0.3)*	0.10 (0.06 to 0.15)**	0.004 (0.001 to 0.007)
Coal coefficient (*a*_1_)^*d*^	–0.12 (–0.25 to 0.01)	0.00005 (–0.006 to 0.006)	0.008 (0.006 to 0.01)*
Previous year coefficient (*d*)^*e*^	0.99 (0.98 to 0.99)*	0.960 (0.958 to 0.962)*	0.953 (0.951 to 0.955)*
LE at birth (years)
Intercept (*a*_0_) in change in LE per year	1.2 (1.0 to 1.4)*	1.6 (1.1 to 2.2)*	–0.36 (–0.84 to 0.13)
Electricity coefficient (*b*_1_)^*d*^	–0.01 (–0.07 to 0.04	0.009 (–0.026 to 0.044)	–0.001 (–0.005 to 0.003)
Coal coefficient (*a*_1_)^*d*^	–0.006 (–0.02 to 0.01)	–0.009 (–0.013 to –0.004)*	–0.002 (–0.004 to 0.001)
Previous year coefficient (*d*)^*e*^	0.988 (0.984 to 0.992)*	0.982 (0.973 to 0.991)*	1.01 (1.00 to 1.02)*
*a*_1_, *b*_1_, and *d* represent the coefficients of the model parameters (as defined in Equation 1). ^*a*^Algeria, Brazil, India, Indonesia, Pakistan, Peru, South Africa, and Turkey. ^*b*^Argentina, Chile, China, Columbia, Greece, Hungary, Mexico, Poland, Portugal, Romania, South Korea, and Thailand. ^*c*^Australia, Austria, Belgium, Bulgaria, Canada, Denmark, Finland, France, Germany, Ireland, Italy, Japan, Netherlands, New Zealand, Norway, Slovak Republic, Spain, Sweden, Switzerland, United Kingdom, and the United States. ^*d*^Unit change in IM or LE per 1,000 kWh/person/year unit change. ^*e*^Unit change in IM or LE as a fraction of previous year’s IM (LE). *p < 0.05.			

## Results

**Figure 2:**
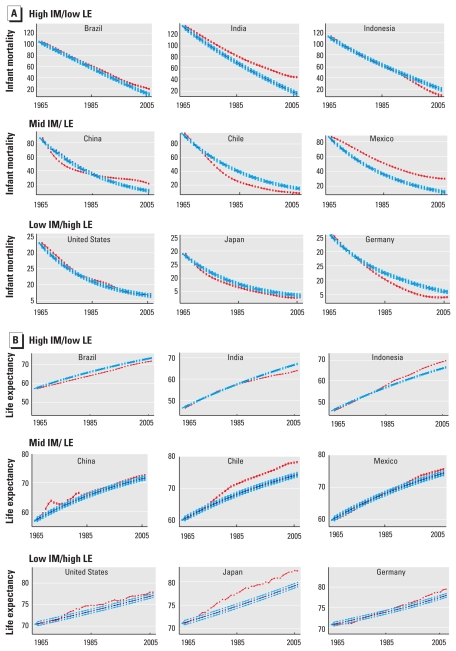
AR model results for IM per 1,000 live births (*A*) and LE at birth (years; *B*) across time, based on rates in 1965 (blue; with 95% confidence intervals), versus observed data (red), for three countries classified in each of the three categories: high IM/low LE (Brazil, India, and Indonesia), mid-IM/LE (China, Chile, and Mexico), and low IM/high LE (United States, Japan, and Germany).

*AR models for low-, mid-, and high-IM/LE countries.* Figure 1 plots composite AR models against IM and LE for the highest population country in each of the three categories (India, China, and the United States). Comparisons of the raw data with the fitted models suggest a good fit to these data (*R*^2^ = 0.66–0.92). Figure 2 presents time-series model results for nine countries, highlighting the differences between countries starting with high IM/low LE in 1965 and countries starting with mid-IM/LE and low IM/high LE. Table 1 presents model parameter values for each group. As expected, the rates of decrease in IM and increase in LE are much lower for countries that began with the lowest IM and the highest LE. For each of these models, the previous year’s coefficients for IM and LE, which can be interpreted as surrogates for overall improvements expected with time (e.g., overall development trajectory that would include education, vaccination rates, health care access, and spending), are important factors in predicting current IM or LE, respectively (Table 1).

The model predicted a significant inverse relationship between electricity consumption and IM for countries with high IM/low LE in 1965. Interestingly, the model estimated a significant positive relationship between electricity consumption and IM for countries with mid-IM/LE and low IM/high LE in 1965. Electricity consumption was not significantly predictive of LE in high-IM/low-LE or low-IM/high-LE countries, although LE was inversely associated with increasing coal consumption in the mid-IM/LE countries. Finally, we found a significant positive association between coal consumption and IM estimated for the low-IM/high-LE countries (Table 1).

These results corroborate previous research (Modi et al. 2005; Wilkinson et al. 2007) that suggested electricity consumption is important for improving overall public health metrics such as IM in countries with high IM, but there appeared to be an adverse impact on IM in countries with mid-IM and low IM. Increased outdoor air pollution, or lifestyle factors associated with higher levels of electricity use (and increased gross domestic product), such as increased chronic disease rates, may explain the significant positive relationship between IM and electricity use in countries with mid-IM and low IM.

Our findings suggest that, controlling for electricity supply, coal consumption negatively affects health. This corroborates a multitude of research (Oak Ridge National Laboratory 1995; Rabl and Spadaro 2006; Spath et al. 1999) on specific health impacts from occupational and environmental exposures related to coal consumption, using broad population-level health metrics over 40 years across 41 different countries. However, this methodology has several limitations, particularly because data sets for potential confounders are unavailable across such a wide geographical space and time period [see Supplemental Material, “Limitations of AR Models” and Table 1 (doi:10.1289/ehp.1002241)]. Therefore, we further explored the relation between energy consumption and health using bottom-up methodologies that apply exposure–response relationships identified for specific health end points associated with energy production (e.g., PM exposure and mortality).

*Comparison with environmental burden of disease reports.* Next, we assessed specific health impacts that may be driving the significant relationships between electricity use, coal consumption, and the broad health metrics of LE and IM noted above. We applied two independent methods for modeling health impacts from environmental exposures related to energy consumption and production. First, we used the WHO environmental burden of disease (EBoD) disability estimates (Ezzati et al. 2004), which are based on a standardized approach for evaluation of health impacts from environmental burdens. For example, the EBoD estimates of the global health impacts of ambient air pollution in 2002 used exposure scenarios that covered major metropolitan areas around the world and a dose–response function from a large, peer-reviewed epidemiological study (Pope et al. 1995). EBoD expresses health impacts in total mortality attributed to the exposure, as well as disability-adjusted life years (DALYs), which incorporates disease states (e.g., asthma attributed to ambient air pollution) in addition to mortality.

In this analysis, we expected that if a relationship exists between electricity consumption and health, then the electricity and coal consumption in 2002 would correlate with the EBoD estimates of DALYs lost because of deficient water and sanitation, indoor air pollution, and outdoor air pollution. Electricity consumption per capita is negatively correlated with estimated DALYs lost because of the three environmental determinants of disease, both combined and individually (water and sanitation, indoor air pollution, and outdoor air pollution), for all countries (Table 2). This result indicates electricity use is associated with better health. When results are stratified by IM/LE classification, negative correlations with DALYs lost because of water and sanitation and indoor air pollution are higher for high-IM/low-LE and mid-IM/LE countries than for low-IM/high-LE countries. These trends are consistent with the hypothesis that access to electricity contributes to reducing the disease burden of diarrheal and acute lower respiratory infections (end points measured in the water and sanitation and air pollution EBoD studies, respectively). This could be explained by increased access to clean water associated with centralized power and reduced indoor air pollution related to reduced reliance on biomass or coal burning for cooking and heating.

**Table 2:** Correlation coefficients (*r*) and *p*-values for electricity or coal consumption (per capita) and the EBoD DALYs associated with water and sanitation (water), indoor air pollution (indoor), and outdoor air pollution (outdoor) in 2002 across 41 countries.*a*

EBoD category	Electricity		Coal	
	*r*	*p*-Value	*r*	*p*-Value
Water
All countries	–0.418	0.007	–0.215	0.178
High IM/low LE	–0.667	0.071	–0.375	0.360
Mid-IM/LE	–0.763	0.004	–0.513	0.088
Low IM/high LE	–0.328	0.147	–0.215	0.349
Indoor
All countries	–0.332	0.034	–0.242	0.128
High IM/low LE	–0.609	0.109	–0.252	0.548
Mid-IM/LE	–0.583	0.047	–0.199	0.536
Low IM/high LE	–0.355	0.114	–0.243	0.290
Outdoor
All countries	–0.437	0.004	–0.161	0.316
High IM/low LE	–0.122	0.774	0.424	0.295
Mid-IM/LE	–0.014	0.966	0.040	0.902
Low IM/high LE	–0.394	0.078	0.013	0.955
Water and indoor
All countries	–0.395	0.011	–0.231	0.147
High IM (low LE)	–0.651	0.080	–0.375	0.360
Mid-IM (mid-LE)	–0.726	0.008	–0.407	0.189
Low IM (high LE)	–0.334	0.139	–0.222	0.334
Water and indoor and outdoor
All countries	–0.425	0.006	–0.238	0.135
High IM/low LE	–0.639	0.089	–0.338	0.413
Mid-IM/LE	–0.778	0.003	–0.413	0.182
Low IM/high LE	–0.436	0.048	–0.194	0.400
^*a*^The 41 countries included in this analysis are listed in Table 1 notes.				

*Comparison with the GAINS model of health impacts from coal-fired power stations.* To assess the impact of coal-fired power generation on mortality more closely, we applied the GAINS model (Amann et al. 2008) to estimate air pollutant emissions from coal-fired power plants, consequent human exposure to PM, and the potential life-shortening effect of this exposure. Table 3 shows estimated effects of total emissions of particulate matter with aerodynamic diameter ≤ 10 μm (PM_10_) from coal-fired power stations on the average YLL in the European Union, India, and China. Relationships between PM emissions and YLL based on the GAINS model were similar across the regions. The GAINS model prediction was similar to the AR model prediction of YLL according to PM_10_ emissions for the European Union but was higher than the AR-based estimate for India and lower than that for China. However, for all three predictions, the confidence intervals of the AR model encompassed the GAINS predicted point estimate. GAINS- and AR-based estimates may also differ because the GAINS model estimates YLL among persons > 30 years of age only, whereas the AR time-series analysis estimates changes in LE from birth and therefore incorporates impacts on mortality at all ages.

**Table 3:** Estimated impact, by region, of coal-fired power stations on PM emissions and YLL over the lifetime of a cohort of adults > 30 years of age: GAINS model versus AR model.

Region	Total PM_10_ emissions (kilotons)	Predicted average YLL per capita (GAINS)	Predicted average YLL (95% CI) per capita (AR model, Table 1)^*a*^
European Union (EU-27)	1,000	0.5	0.82 (–0.45 to 2.1)
India	7,000	2.5	0.72 (–1.60 to 3.03)
China	10,000	3.5	6.30 (3.06 to 9.53)
CI, confidence interval. ^*a*^Translation of the coal consumption coefficient (*a*_1_) into units comparable to YLL per capita is described in “Materials and Methods” and entailed multiplying by estimates of average coal consumption and LE.				

## Discussion

The International Energy Agency projects a 50% increase in global energy demand in the next 20 years, driven largely by the fast-growing economies of China and India [International Energy Agency (IEA) 2007]. Increased power generation accounts for approximately half of this increase, and transport for a further one-fifth. Currently, coal is the dominant fuel used for power generation (> 40%), and in the absence of policy changes, its share will rise, given trends in recent years, particularly in China and India (IEA 2007).

This analysis attempts to clarify the independent effects of electricity and coal consumption on global health. We have examined historical time-series trends and compared the results with two health-impact modeling approaches, demonstrating consistency in relationships identified across these independent methods. Several factors are important to consider when comparing the “bottom-up” GAINS model to the “top-down” time-series analysis. The “bottom-up” GAINS methodology uses complex models to estimate PM_10_ emissions from coal-fired power plants, population-level PM_10_ exposures resulting from these emissions, and the impact of these exposures on LE (YLL) among those > 30 years of age. In contrast, our “top-down” AR time-series analysis incorporated historical data on LE, IM, electricity use, and coal consumption over a 40-year period to estimate the impact of coal consumption (vs. PM_10_ emissions due to coal consumption) on LE from birth and IM across 41 countries that differ in geography, economy, and culture. Direct comparisons between the two approaches are complicated by differences in their data sources, assumptions, and estimated outcomes and exposures. Nonetheless, results based on these two distinctly different approaches both support the hypothesis that coal consumption results in quantifiable health impacts.

Under the assumption that historical trends hold relevance today, the results of these health-impact models can inform climate change mitigation strategies. For example, time-series modeling suggests that electricity consumption is significantly associated with improved health only in countries with IM > 100/1,000 live births, whereas in countries with IM < 100/1,000 live births in 1965 the analysis suggests that electricity consumption is associated with increased IM. At present, national IM rates are < 100/1,000 live births in all 41 countries. However, as a recent climate change mitigation strategy highlights (Chakravarty et al. 2009), it is critical to take into account the distribution of electricity use and health status within countries to further define subpopulations that may benefit from increased access to electricity.

Electricity coefficients are significant for models of IM but not for LE. We hypothesize this may be due to the greater vulnerability of infants in impoverished circumstances to environmental threats (e.g., contaminated water and poor sanitation), which tend to be mitigated with access to a reliable electricity source in high-IM/low-LE circumstances and greater susceptibility to mortality due to acute lower respiratory infections associated with air pollution in the mid-IM/LE and low-IM/high-LE case. Impacts on IM are more immediate than are impacts on LE; therefore, they are more easily captured by the regression model, and differences in statistical power due to the smaller magnitude of the LE estimates may also play a role in this result. Future analysis of specific causes of death in countries where data are available across a sufficient time period would be a good starting point to begin teasing apart these relationships.

Our findings from the analysis of historical trends suggest that, controlling for electricity supply, coal consumption negatively affects health (Table 1), and integrated modeling approaches such as GAINS are consistent with this result. Therefore, the projected increase in use of coal for power generation is a great concern (Holdren and Smith 2000; Markandya and Wilkinson 2007; Markandya et al. 2009). Even with controls to reduce sulfur oxides and PM emissions, coal-burning power plants produce relatively large amounts of air pollution. Also, power generation from coal using current technology is more carbon intensive than is any other energy system.

Results from the present top-down time-series analysis of broad health indicators across 40 years in 41 countries support the conclusions of external costs research—large, unaccounted for health costs are associated with coal consumption. We acknowledge there are limitations in the work reported here, because AR models may not accurately account for unmeasured confounders by using the previous year’s IM (LE) to capture the effect of unspecified variables that vary linearly with time. The present time-series analysis would have been greatly improved if comprehensive data sets were available on several potential explanatory variables, including education level, vaccination rates, and health care access and expenditures.

Application of a standardized method for evaluation of global health impacts related to energy systems will be critical as climate change mitigation strategies are negotiated internationally. The WHO methodology establishes a standardized framework for the quantification of global health impacts that is not based on estimating a monetary value of health impacts (Ezzati et al. 2004). This is critical when using results for international policy development because methods used for the monetization of health impacts pose significant concerns among global health researchers, because it is particularly difficult to determine a monetary value for death or disability that is applicable across nations with vastly different cultures and values (Patz et al. 2007; Smith and Haigler 2008).

In summary, we assess the relationship between electricity use and coal consumption and health through analysis of historical data sets and comparison with exposure response models. Previous large-scale economic analyses have suggested that health costs related to air pollution and climate change are the dominant external costs associated with power generation systems, and our analysis points to ways in which health impacts can be integrated into climate change mitigation and energy policy research. We report consistent results using three different approaches to understanding relations between electricity, coal consumption, and health. Overall, it appears that increased electricity consumption in countries with IM < 100/1,000 births (and LE > 57 years) does not lead to greater health benefits and that coal consumption has significant detrimental health impacts.

## Supplemental Material

(1.4 MB) PDF
