# Erratum: “Estimating the Global Public Health Implications of Electricity and Coal Consumption”

**DOI:** 10.1289/ehp.122-A295

**Published:** 2014-11-01

In Table 3 of “Estimating the Global Public Health Implications of Electricity and Coal Consumption” by Gohlke et al. [Environ Health Perspect 119:821–826 (2011); http://dx.doi.org/10.1289/ehp.1002241], the upper 95% confidence limits for the predicted average years of life lost (YLL) per capita were inadvertently changed to negative numbers. The corrected table is presented below.

**Table 3:** Estimated impact, by region, of coal-fired power stations on PM emissions and YLL over the lifetime of a cohort of adults > 30 years of age: GAINS model versus AR model.

Region	Total PM_10_ emissions (kilotons)	Predicted average YLL per capita (GAINS)	Predicted average YLL (95% CI) per capita (AR model, Table 1)^*a*^
European Union (EU-27)	1,000	0.5	0.82 (–0.45 to 2.1)
India	7,000	2.5	0.72 (–1.60 to 3.03)
China	10,000	3.5	6.30 (3.06 to 9.53)
CI, confidence interval. ^***a***^Translation of the coal consumption coefficient (*a*_1_) into units comparable to YLL per capita is described in “Materials and Methods” and entailed multiplying by estimates of average coal consumption and LE.			

*EHP* regrets the error.

