# Supplementary material for: Estimating the Global Public Health Implications of Electricity and Coal Consumption
Source: Environ Health Perspect. 2011 Feb 21;119(6):821–6. doi: 10.1289/ehp.119-821 (PMC3114817; doi:10.1289/ehp.119-821)
Supplement: (1.4 MB) PDF [file ehp.119-821.s001.pdf]

SUPPLEMENTAL MATERIAL

Estimating the Global Public Health Implications of Electricity and Coal Consumption

Julia M Gohlke, Reuben Thomas, Alistair Woodward, Diarmid Campbell-Lendrum, Annette Prüss-Üstün, Simon Hales, Christopher J Portier

Table of Contents

Methods-Data Description**... 3**

Methods-AR Model Description **.. 3**

Methods-GAINS Model Description**.. 5**

Limitations of AR Models.. 5

Supplemental Table 1: Time period of datasets available on explanatory variables in the AR models. **................. 6**

Supplemental Figure 1: Infant mortality rates between 1965 and 2005 across 41 countries...................................7

Supplemental Figure 2: Life expectancy at birth between 1965 and 2005 across 41 countries...............................**8**

Supplemental Figure 3: Per capita electricity consumption between 1965 and 2005 across 41 countries..............**9**

Supplemental Figure 4: Per capita coal consumption between 1965 and 2005 over 41 countries.........................**10**

Supplemental Figure 5: Individual AR model results for IM across 28 of the 41 countries analyzed. **............... 11**

References..12

Methods-Data Description

When small gaps in data were present (< 5 years), these were filled using linear interpolation between the two nearest data points in time. In the case of IM, data were only available in 10 year increments from 1960 to 1990 and 5 year increments from 1990 to 2005. The missing data were estimated by applying the spline function in Matlab 2008a to interpolate between the data points. Particularly for models using the IM datasets, the non-independence of the interpolated datapoints leads to greater uncertainty in terms of the confidence intervals of the model results. Data for several other factors that may predict LE or IM were sought during the preliminary stages of this project. However, none were available for the time period under analysis, as many relevant statistics on education level, vaccination rates, improved water sources, female literacy rates, and health care access and spending are not available across all 41 countries until the 1990s (See Supplemental Material, Table 1). This limitation led us to choose the autoregressive instead of a full multivariate regression methodology, which would require datasets on all potential explanatory variables. The autoregressive method is described in more detail below.

Methods-AR Model Description

To separate the dependencies of LE or IM solely due to coal and electricity consumption patterns, at each time point until time t, $Q(t)$, with the dependencies due to all other reasons, $P(t)$ that can be modelled by its exponential functional form and those associated with the errors of predicting LE or IM at each time point until time t, that could not be captured by either $P(t)$ or $Q(t)$ at each time point until time t:

$$
\begin{aligned}
y(t) &= P(t) + Q(t) + E(t) \qquad [2] \\
P(t) &= a_0(1 + d + d^2 + \ldots + d^{t-1}) + y(0)d^t \\
&\approx a_0 t + y(0)\text{: if d} \approx 1 \\
Q(t) &= a_1 u_1(t) + b_1 u_2(t) + \sum_{i=1}^{(t-1)} d^i (a_1 u_1(t-i) + b_1 u_2(t-i)) \\
&\approx a_1 \sum_{i=0}^{(t-1)} u_1(t-i) + b_1 \sum_{i=0}^{(t-1)} u_2(t-i)\text{: if d} \approx 1 \\
E(t) &= e(t) + \sum_{i=1}^{(t-1)} d^i e(t-i) \\
&\approx \sum_{i=0}^{(t-1)} e(t-i)\text{: if d} \approx 1
\end{aligned}
$$

where $y(0)$ is the LE or IM in the initial year, 1965. The parameter d can be interpreted as the influence of the past values of LE or IM and past values of coal and electricity consumption on the current observed LE or IM values. Observation of the estimates of the parameters in the model provided in Table 1 indicates that the parameter d is approximately 1 for all the different model fits considered. This allows us to approximate the functions $P(t)$, $Q(t)$ and $E(t)$ as shown in the equations above and hence provide better interpretations of the remaining parameters of the model. Note this approximation is made just to better illustrate the significances of the parameters. The actual values of the parameters were estimated using the exact form of the model. The parameters a_1, b_1 represent the effect of the coal consumption per capita at all time points until year t and electricity consumption per capita at all time points until year t, respectively on the LE or IM in year t. a_0 is approximately the linear rate of increase of LE or decrease of IM with time that can be interpreted as a surrogate for yearly improvements in life expectancies and IM due to factors such as economic development, access to effective health care, and technological improvements. Generation of individual country models showed a_0 varied across the countries based on LE and IM in 1965 such that countries with high IM and low LE had much larger values for a_0 than for countries starting with relatively low IM and high LE. Under the assumption that impacts of environmental exposures on IM and LE vary widely based on this parameter, we developed three separate composite models based on IM and LE in 1965.

Methods-GAINS Model Description

In the GAINS model, a linked sequence of calculations leads to estimates of health impact. First, the effects of energy sources and policies on air pollution emissions are estimated. The calculation is based on emission factors and control technologies for specific activities such as electricity generation. Resulting emission inventories for air pollutants along with weather data are used as inputs to a global-regional chemistry transport model. The atmospheric model is used to estimate the functional relationships between emissions of air pollutants in a given (source) region and atmospheric concentrations in other (receptor) regions. In turn, these results are used to derive spatially explicit estimates of air pollutant concentrations. The air pollution concentration estimates, combined with population distribution data, provide exposure estimates. These, along with baseline mortality data and external dose-response estimates, are used to estimate health impacts. A recent analysis of climate induced impacts to air pollution presents a detailed analysis of the GAINS model (Markandya et al. 2009). The most important assumptions required by the model, and their possible effects are described in detail elsewhere (Amann et al. 2008). Broadly, this integrated assessment model draws on emission inventories based on expected values for fuel quality, relies on accuracy of statistical information on economic activities, assumes linearity in the atmospheric dispersion calculations and depends on expected values of conversion and deposition rates describing chemical and physical processes, as well as the reliability of epidemiological data describing the health impact of a particular exposure.

Limitations of AR Models

In the analysis of historical trend data, one must be concerned with confounders. For example, electricity or coal consumption could serve as a surrogate for wealth, which may be the ultimate reason for increased health through any of a host of mechanisms such as increased availability and access to health care, increased vaccination rates, and increased education levels. It is noted that incorporating the linear time trend (a_0) as well as previous year's IM (LE) captures the effects of unspecified variables that vary linearly with time. The AR methodology employed here is commonly used to account for unmeasured confounders (Kale et al. 2004; Kovats et al. 2004; Levine et al. 2001) and see also discussion in methods section). The uniqueness of the present study is analysis of

datasets across 41 different countries over a time period of 40 years. Data for several other factors that may predict LE or IM were sought during the preliminary stages of this project, but none were available for the time period under analysis, as many statistics on education and vaccination rates have only recently been collected across all the countries included in this study (See Supplemental Material, Table 1). It is not known whether specific potential confounders such as education or vaccination levels are being implicitly modelled in the AR terms, but the model does fit well to the data (as per the R^2 values of regression) and is not systematically over or under predicting. Some well documented non-linearities, such as the effects of the AIDS epidemic in South Africa and the transition from communism in Eastern Europe are evident in the individual time series models (See Supplemental Material, Figure 5).

The a_0 parameter could be considered to be confounded with the coal and electricity consumption parameters, a_1 and b_1 if the cumulative coal consumption and cumulative electricity consumption also varied linearly over time. However this is not the case as only 2/41 countries (Romania and Bulgaria) had insignificant (at the 95% confidence level) quadratic terms for coal consumption and 1/41 (Romania) for electricity consumption.

Supplemental Table 1. Datasets available on potential explanatory variables are only available for a fraction of the time period analyzed in the time-series analysis.

Factor	***Years covered***	***Source***
% of females completing primary education; family planning education	1995-present	World Development Indicators, World Bank
Female employment rate	1991-present	International Labor Organization
% of government spending on health (or per capita spending)	1995-present	World Health Organization
Improved water source	1990-present	World Development Indicators, World Bank
Immunization rate	1990-present	World Health Organization
Female Literacy rate	1980-present	UNESCO

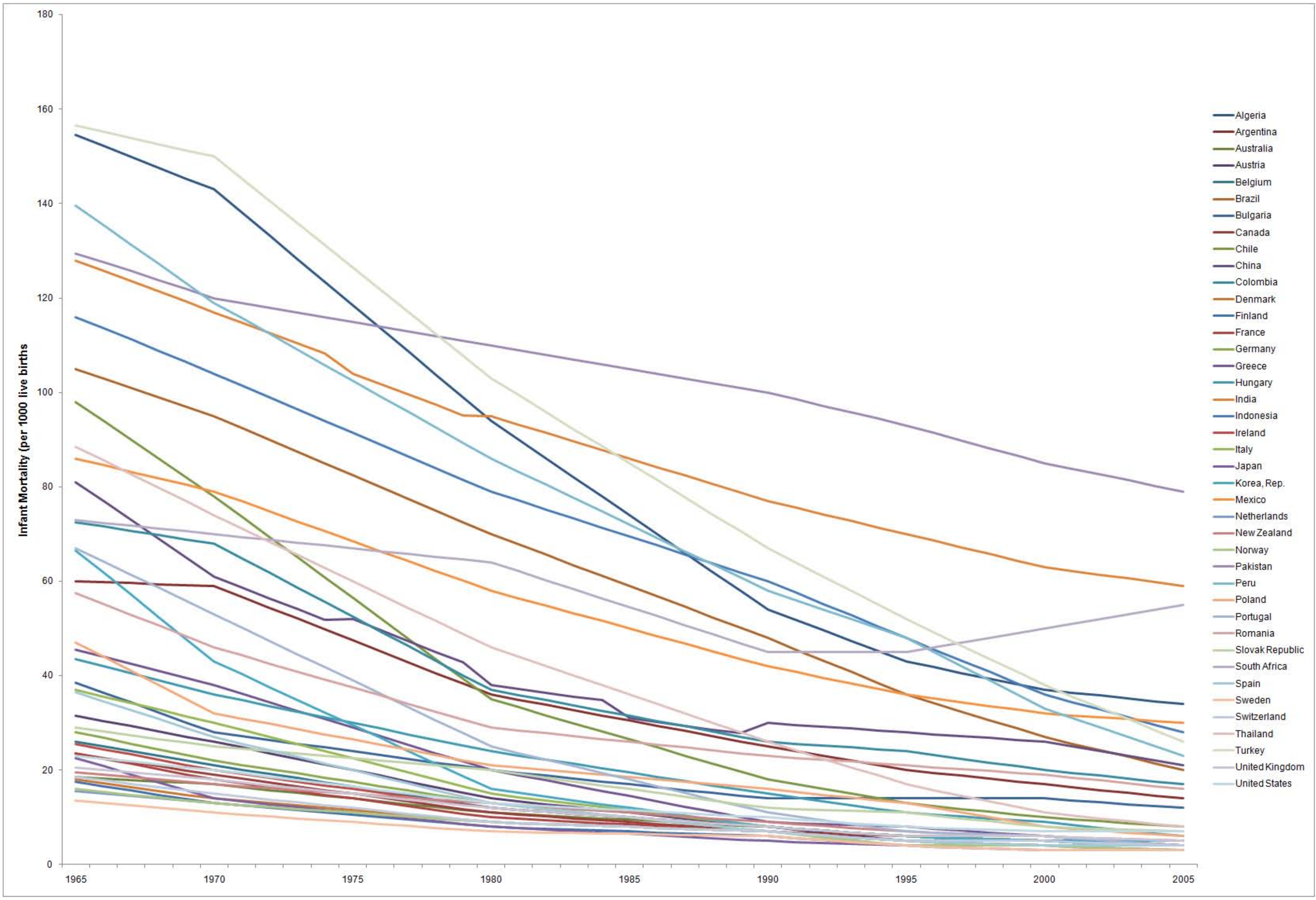

Supplemental Figure 1: Infant mortality rates between 1965 and 2005 across 41 countries. Data downloaded from gapminder.org (Rosling 2009).

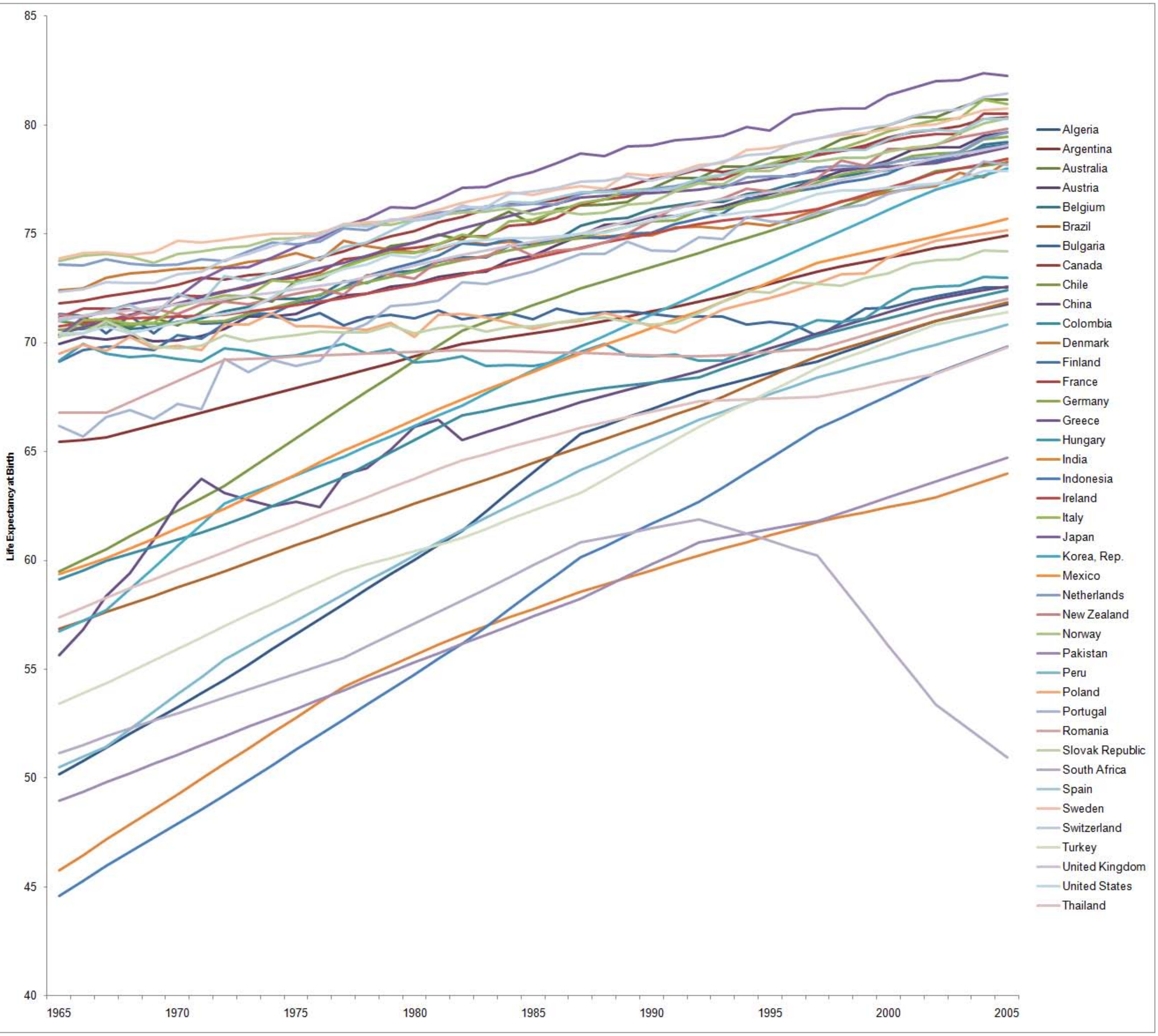

Supplemental Figure 2: Life expectancy at birth between 1965 and 2005 across 41 countries. Data downloaded from gapminder.org (Rosling 2009).

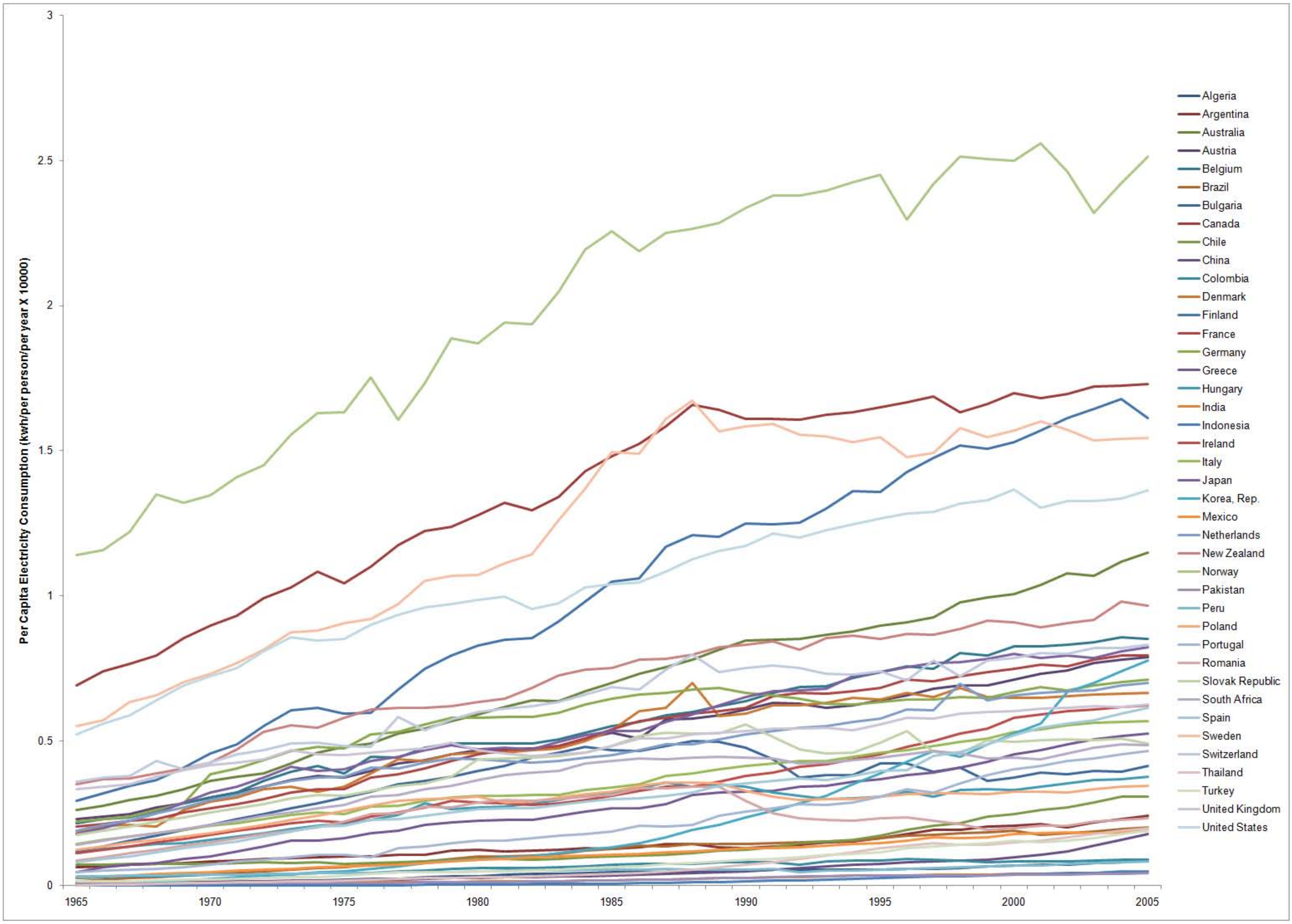

Supplemental Figure 3: Per capita electricity consumption between 1965 and 2005 across 41 countries. Data downloaded from gapminder.org (Rosling 2009).

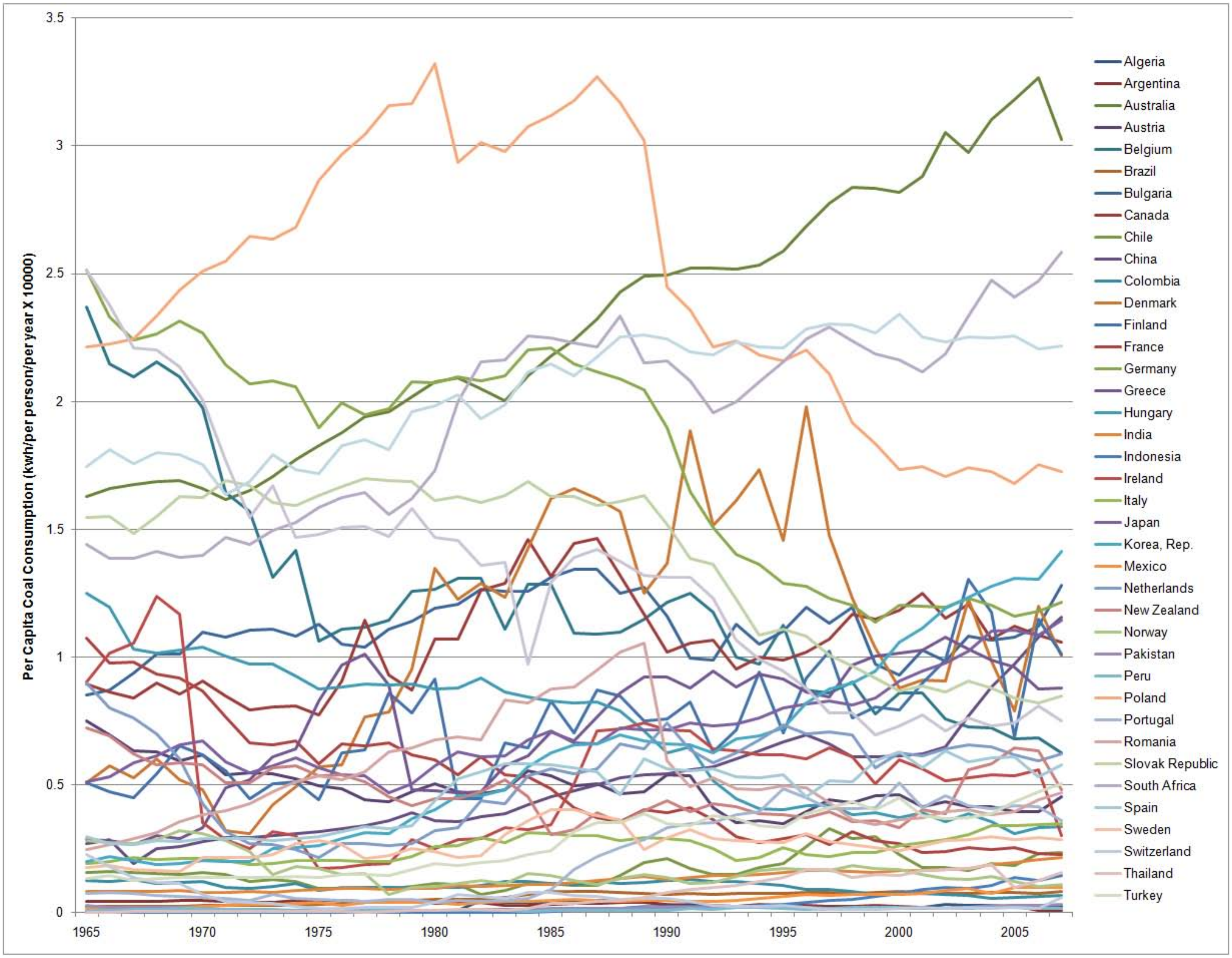

Supplemental Figure 4: Per capita coal consumption between 1965 and 2005 over 41 countries. Data downloaded from gapminder.org (Rosling 2009).

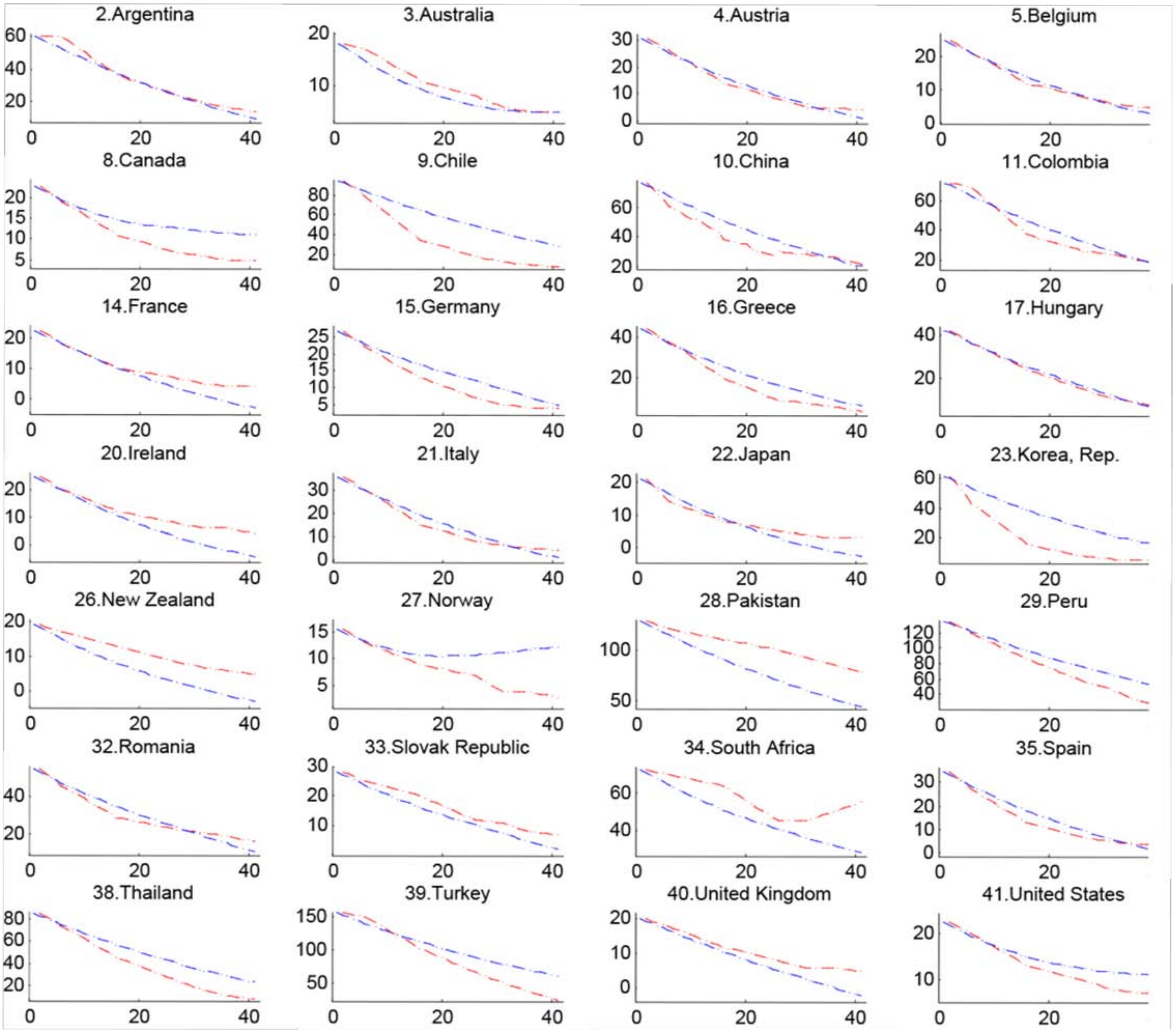

Supplemental Figure 5. Individual autoregressive model results for infant mortality (IM) across 28 of the 41 countries analyzed. IM (per 1000 births) for individual countries is plotted across the time period in red. Results of the model described by Equation 1 for individual countries are plotted in blue. Note consistency in overall IM reduction rates across models with initial low, mid, and high starting IM. Results are alphabetically sorted, and every 5 and 6th country (the 1, 6, 7, 12, 13, 18, 19, 24, 25, 30, 31, 36, and 37th) are not presented due to graphical space limitations.

REFERENCES

Amann M, Bertok I, Borken J, Chambers A, Cofala J, Dentener F, et al. 2008. GAINS-ASIA. A tool to combat air pollution and climate change simultaneously. International Institute for Applied Systems Analysis (IIASA) Laxenburg, Austria.

Kale PL, Andreozzi VL, Nobre FF. 2004. Time series analysis of deaths due to diarrhoea in children in Rio de Janeiro, Brazil, 1980-1998. Journal of health, population, and nutrition 22(1): 27-33.

Kovats RS, Hajat S, Wilkinson P. 2004. Contrasting patterns of mortality and hospital admissions during hot weather and heat waves in Greater London, UK. Occup Environ Med 61(11): 893-898.

Levine RS, Foster JE, Fullilove RE, Fullilove MT, Briggs NC, Hull PC, et al. 2001. Black-white inequalities in mortality and life expectancy, 1933-1999: implications for healthy people 2010. Public Health Rep 116(5): 474-483.

Markandya A, Armstrong BG, Hales S, Chiabai A, Criqui P, Mima S, et al. 2009. Public health benefits of strategies to reduce greenhouse-gas emissions: low-carbon electricity generation. Lancet 374(9706): 2006-2015.

Rosling H. 2009. Gapminder World. http://www.gapminder.org/. [accessed Dec. 2010].
